# Effects of HIV related stigma on the lives of persons living with HIV

**DOI:** 10.1186/1471-2334-12-S1-P52

**Published:** 2012-05-04

**Authors:** SJ Parameswari, N Jayapoorani

**Affiliations:** 1 Avinashilingam Institute for Home Science and Higher Education for Women, University, Coimbatore-641 043, India

## Background

Persons living with HIV are stigmatized throughout the world in varying degrees. PLHIVs experience stigma in two forms – internal and external stigma. Due to internal stigma, PLHIVs isolate themselves from the community and they do not even access essential health care services. Due to external stigma, PLHIVs are rejected by their loved ones and their community, unfairly treated in the workplace and are denied access to health services. The present study made an attempt to bring out the effects of HIV related stigma on the lives of PLHIVs in the Nilgiris.

## Methods

To measure the existence of internal and external stigma 180 PLHIVs were selected. Various qualitative research methods such as focus group discussions and case study were adopted to gather necessary data. Focus group discussions were conducted with 47 PLHIVs (11 male and 36 female). Case study highlights the in depth situation of the PLHIVs, hence eight PLHIVs were interviewed and gathered needed information with their permission.

## Results

Ninety four percent of respondents mentioned about the feelings of rejection. Ninety two per cent PLHIVs conversed about the guilty feeling of being a PLHIV. Ninety four percent of selected PLHIVs mentioned that they were emotionally affected due to the discriminatory activities of their own family members. Children of PLHIV parents experienced discriminatory practices in the school regardless of their HIV status.

## Conclusion

The qualitative data obtained in this study substantiate the stigmatizing and discriminatory experiences such as denial of house for rent, denial of property and marital conflicts etc.

